# Superiority of a Representative MRI Flow Waveform over Doppler Ultrasound for Aortic Wave Reflection Assessment in Children and Adolescents With/Without a History of Heart Disease

**DOI:** 10.1007/s10439-023-03339-2

**Published:** 2023-08-10

**Authors:** Jonathan P. Mynard, Remi Kowalski, Hilary A. Harrington, Avinash Kondiboyina, Joseph J. Smolich, Michael M. H. Cheung

**Affiliations:** 1 Heart Research, Murdoch Children’s Research Institute, 50 Flemington Road, Parkville, VIC 3052 Australia; 2 Department of Paediatrics, University of Melbourne, Parkville VIC, Australia; 3 Department of Biomedical Engineering, University of Melbourne, Parkville VIC, Australia; 4 Department of Cardiology, Royal Children’s Hospital, Parkville VIC, Australia

**Keywords:** Pulse analysis, Blood pressure, Wave reflection, Children, Adolescents, Congenital heart disease

## Abstract

Wave separation analysis (WSA) reveals the impact of forward- and backward-running waves on the arterial pressure pulse, but the calculations require a flow waveform. This study investigated (1) the variability of the ascending aortic flow waveform in children and adolescents with/without a childhood heart disease history (CHD); (2) the accuracy of WSA obtained with a representative flow waveform (RepFlow), compared with the triangulation method and published ultrasound-derived adult representative flow; (3) the impact of limitations in Doppler ultrasound on WSA; and (4) generalizability of results to adults with a history of CHD. Phase contrast MRI was performed in youth without (*n* = 45, Group 1, 10–19 years) and with CHD (*n* = 79, Group 2, 7–18 years), and adults with CHD history (*n* = 29, Group 3, 19–59 years). Segmented aortic cross-sectional area was used as a surrogate for the central pressure waveform in WSA. A subject-specific virtual Doppler ultrasound was performed on MRI data by extracting velocities from a sample volume. Time/amplitude-normalized ascending aortic flow waveforms were highly consistent amongst all groups. WSA with RepFlow therefore yielded errors < 10% in all groups for reflected wave magnitude and return time. Absolute errors were typically 1.5–3 times greater with other methods, including subject-specific (best-case/virtual) Doppler ultrasound, for which velocity profile skewing introduced waveform errors. Our data suggest that RepFlow is the optimal approach for pressure-only WSA in children and adolescents with/without CHD, as well as adults with CHD history, and may even be more accurate than subject-specific Doppler ultrasound in the ascending aorta.

**Supplementary Information:**

The online version contains supplementary material available at 10.1007/s10439-023-03339-2.

## Introduction

High blood pressure (BP) and early vascular ageing in children and adolescents are significant health concerns, given an increasing prevalence of paediatric hypertension [1], and consistently observed associations between high BP and end organ damage (such as arterial stiffening and microvascular remodeling) in this age group [2–5]. Moreover, young people with a history of congenital or childhood heart disease (CHD) are more likely to exhibit a range of vascular and haemodynamic abnormalities [6–11], related to higher rates of hypertension, cerebrovascular disease, and premature mortality in some sub-groups [12–14]. Thus, there is a need to establish accessible and robust techniques for characterization of early cardiovascular risk in young people.

Wave separation analysis quantifies the contribution of forward- and backward-running waves to pulsatile haemodynamics [15], providing more detailed information about ventriculo–arterial interactions than systolic and diastolic blood pressure values. Forward waves are produced by the acceleration and deceleration of blood by active ventricular dynamics, whilst backward waves arise from wave reflection in the arterial network. These backward waves are also re-reflected at the ventricle, making an additional contribution to the forward wave [16–18]. Importantly, the extent to which wave reflection indicates the presence of arterial stiffening/disease, elevated ventricular load, and risk of events such as heart failure, depends on the magnitude of the reflected waves as well as their return time [19–24].

One of the limiting factors for performing conventional wave separation analysis is that both a pressure and flow waveform are required [15]. Although a central pressure signal can be obtained inexpensively with techniques such as carotid tonometry, radial tonometry, or cuff volume plethysmography (with mathematical transformation, where required, to obtain central waveforms), measurement of an ascending aortic flow waveform non-invasively requires techniques such as ultrasound or magnetic resonance imaging (MRI), which are more expensive and require expert operators. In large population studies, acquisition of ascending aortic flow waveforms is rarely feasible.

For these reasons, a number of techniques have been explored to estimate or synthesize ascending aortic flow waveforms for use in wave separation analysis. The reasoning is that, unlike the pressure waveform, the ascending aortic flow waveform has a relatively consistent shape in most individuals, as it is less affected by wave reflection [25]. Synthesized or representative flow waveforms need not be calibrated because indices such as reflection magnitude and return time depend only on the shape (but not the amplitude) of the flow waveform [26, 27].

The first such technique was described by Westerhof et al. [27] who reported that a simple triangular flow waveform resulted in good correlations for reflection magnitude (*R*^2^ of 0.8–0.9) when compared with measured high fidelity waveforms. Kips et al. [28] subsequently reported lower correlations with the triangular waveform (*R*^2^ = 0.55), but better results with an averaged physiological flow waveform obtained from ultrasound in adults (*R*^2^ = 0.74). Other investigators have proposed more personalized approximation techniques, with Hametner et al. [29] using a model-based approach and Shenouda et al. [30] using a heuristic method to generate a realistic, synthesized waveform using fiducial points from the individual-specific pressure waveform; both methods yielded estimates of reflection indices with errors less than 10%.

These prior studies have all investigated pressure-only wave separation techniques in adults. Aside from one recent study in healthy 14–19 year-olds [31], no studies have investigated the use of synthesized or representative flow waveforms for wave separation in children or adolescents, nor in young people or adults with a history of CHD. The latter is relevant because abnormalities in heart function or arterial properties could have an effect on the aortic flow waveform. For example, whether a representative flow waveform (obtained by averaging waveforms from healthy individuals) could be generalized to CHD patient groups is unclear.

Finally, most prior studies investigating these techniques used Doppler ultrasound as the reference method for measuring aortic flow waveforms [28, 29, 31, 32]. However, this technique involves several sources of error, including (1) placement of a sample volume in the centre of the vessel (thus not capturing the full cross-sectional velocity profile), (2) the use of an envelope-tracing technique that involves following peak velocity rather than the cross-sectional mean velocity (the former being sensitive to velocity profile skewing, the latter more closely representing volumetric flow), and related to this, (3) an inability to accurately capture flow reversal around the time of valve closure [33, 34].

The first aim of this study was, therefore, to evaluate the degree of variability in ascending aortic flow waveform morphology in children and adolescents without (Group 1) and with (Group 2) a history of CHD, utilizing gold-standard flow data from phase contrast magnetic resonance imaging (PCMRI). Aim 2 was to assess the accuracy of wave reflection indices in these groups, when derived via a representative (averaged) flow waveform obtained from Group 1. Aim 3 was to evaluate the accuracy of the triangulation method and ultrasound-derived representative adult flow waveform reported by Kips et al. [28]. Aim 4 was to evaluate the impact of the limitations of Doppler ultrasound on reflection indices—specifically, velocity profile skewing and limited sample volume coverage—by performing a virtual Doppler ultrasound using PCMRI data. Finally, these aims were also extended to a group of adults with a history of CHD (age 19–59 years, Group 3).

## Materials and Methods

This study was conducted in accordance with the Declaration of Helsinki and was approved by the Human Research Ethics Committee of the Royal Children’s Hospital, Melbourne, Australia.

### Study Participants

Data were acquired from routine cardiac MRI, with patients divided into three groups. *Group 1* (*n* = 45) consisted of children and adolescents aged 10-18 years with normal left ventricular structure and function, and no abnormalities of the aorta. *Group 2* (*n* = 79) comprised paediatric patients with a history of CHD involving known or potential effects on the left ventricle and/or systemic arteries. *Group 3* (*n* = 29) comprised adults with a history of CHD. See Supplemental Material for details.

### Study Protocol

Blood flow in the ascending aorta (at the level of the right pulmonary artery) was obtained from high temporal resolution PCMRI, taking care to ensure perpendicular alignment with the ascending aorta in two orthogonal planes. Scans were performed on an Aera 1.5 Tesla MRI machine (Siemens, Erlangen, Germany) using a segmented 2D phase contrast gradient echo sequence, with a 320 mm field of view, 6 mm slice thickness, 3.38 ms echo time, flip angle of 20°, and a repetition time of 22.8 ms. Two segments were acquired per heartbeat, with interleaved sampling giving a calculated temporal resolution of 128 phases per cardiac cycle. LV volumes and mass were obtained from cine-SSFP sequences planned perpendicular to the axis of the ventricular septum with a slice thickness of 7 mm, with analysis performed using CVI42 software (Circle Cardiovascular Imaging, Calgary, Canada).

### Image Segmentation

Volumetric flow (*Q*) and cross-sectional area (*A*) waveforms were obtained from PCMRI via semi-automated segmentation of the aortic blood-wall boundary, using a custom in-house program written in MATLAB (R2020b, The MathWorks Inc., Natick, Massachusetts); the details of which are described in the Supplemental Material.

### Representative Flow Waveform

A representative flow waveform was generated using data from Group 1 (children/adolescents with normal left ventricle and aorta). Individual flow waveforms were interpolated to 1000 points, amplitude-normalized to a peak of 1.0, and time-normalized to (1) the time at which the initial flow upstroke reached 50% of the peak value (which is insensitive to slight shape variations in the foot), and (2) end-systole, defined as the local minimum following the peak. The population-derived representative flow waveform was obtained by averaging the normalized waveforms and smoothing with a Savitzky-Golay filter spanning 0.125 normalized time units. Finally, to enforce zero flow during the latter part of diastole, the representative waveform was multiplied with a weighting function that had three segments: (1) a value of 1.0 during systole and until flow returned to zero after the end-systolic minimum, (2) a linear ramp from 1.0 to 0.0 over a period of 0.6 normalized time units, and (3) 0.0 thereafter.

The resulting representative flow waveform was de-normalized for use in wave separation analysis in a given individual by aligning the 50% upstroke time and end-systolic time to the respective times detected from the measured patient-specific flow waveform.

### Kips Flow Waveform

In addition to the representative flow waveform obtained from children and adolescents, we also evaluated the performance of the representative waveform obtained in adults via ultrasound and reported by Kips et al. [28] The waveform was digitized from Figure S2 in that reference and was de-normalized in time as described above for RepFlow.

### Triangular Flow Waveform

A synthesized triangular flow waveform, as proposed by Westerhof et al. [27] was also evaluated. Following, Kips et al. [28] the base of the triangle spanned from the onset of the flow upstroke to the end-systolic minima, as this avoided any error introduced by estimating these time points from the pressure waveform. The timing of the triangle apex was determined by three approaches: (1) fixed at 30% of ejection time [27], (“Tri30”); (2) fixed at 25% of ejection time (close to the average time of peak flow in Group 1 in the present study, “Tri25”); and (3) the time of true peak flow for each individual (i.e. the theoretical ‘ideal’ timing, “TriPeak”). Since all of these methods yielded very similar results, for brevity we only present data for Tri25 (referred to as “Triangle” in figures/tables), which was marginally superior to Tri30 and TriPeak based on visual inspection of the data.

### Virtual Doppler Ultrasound

**Fig. 1:**
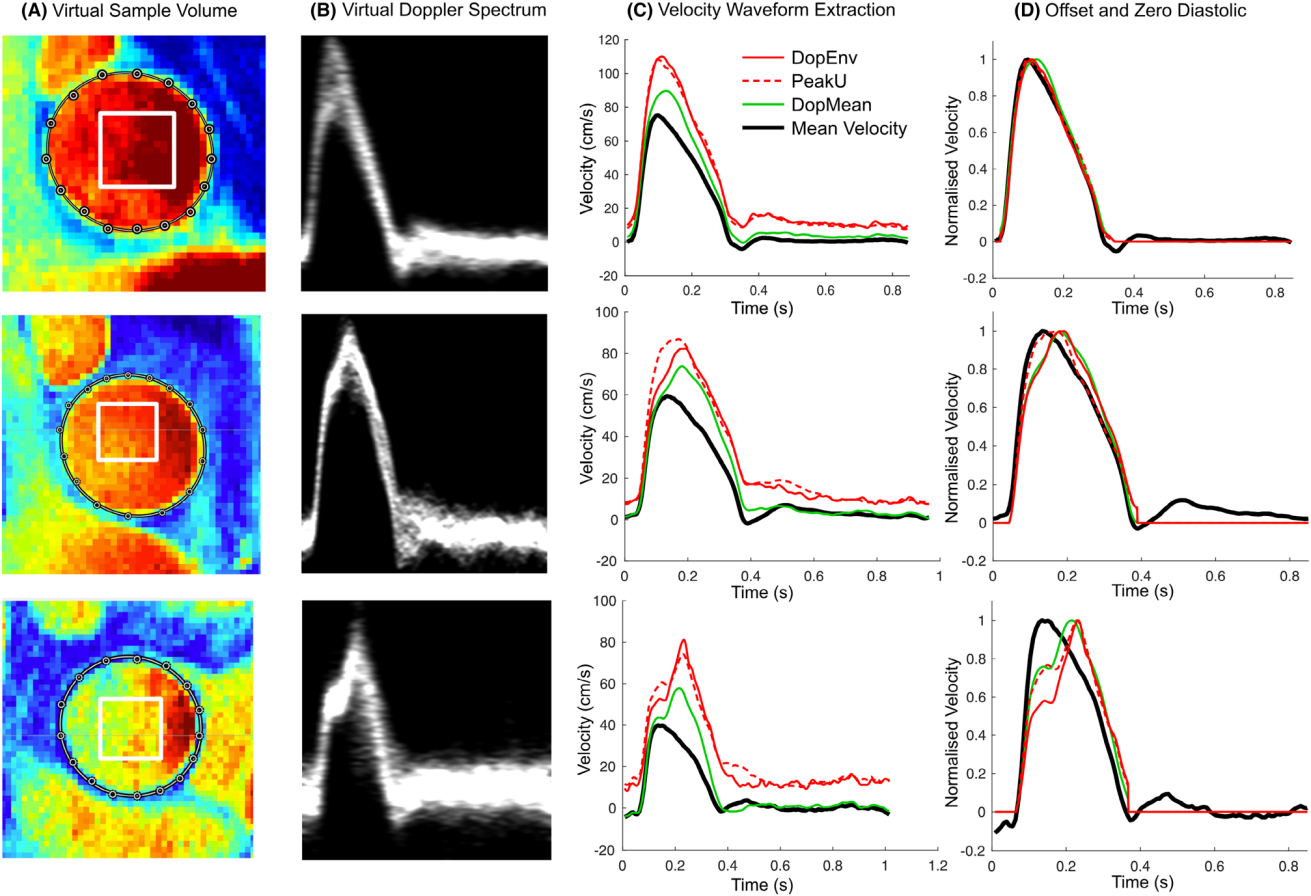
Virtual Doppler ultrasound analysis, showing examples where the virtual Doppler waveforms demonstrated good agreement (top panels), moderate agreement (middle panels) and poor agreement (bottom panels) with the true mean velocity. See text for further explanation. *DopEnv* 95th percentile velocity within the virtual Doppler sample volume, *DopMean* mean velocity within the sample volume, *PeakU* 95th percentile velocity within the whole lumen (note that PeakU can be less than DopEnv due to the use of 95th percentiles).

A square sample volume covering 50% of the minimum effective diameter was placed at the time-averaged centroid location of the lumen obtained from PCRMI (Fig. 1A). Velocities extracted from this sample volume were used to generate a virtual Doppler spectrum (Fig. 1B). The Doppler Envelope waveform (“DopEnv”) was obtained as the instantaneous 95th percentile of the velocity within the sample volume. This was compared with the 95th percentile from the entire lumen (“PeakU”) which represents an ideal scenario in which the Doppler sample volume perfectly covers the entire lumen. The Doppler Mean waveform was obtained as the instantaneous mean velocity within the sample volume (“DopMean”). DopEnv and PeakU tended to have non-zero values during diastole (Fig. 1C), and therefore, these waveforms (as well as DopMean) were offset to a zero end-diastolic value at beat onset and then zeroed during diastole (Fig. 1D). Resulting waveforms used for analysis are compared with the true mean velocity waveform after normalization in Fig. 1D, which shows one example where the resulting waveforms were all similar (top panels), and two examples where differences in the waveforms were caused by a skewed velocity profile (middle/bottom panels). All examples shown in Fig. 1 are from Group 1 (normal LV/aorta).

### Wave Separation

Wave separation was performed using cross-sectional area as an uncalibrated surrogate of pressure (*P*), and flow (*Q*) obtained from (1) patient-specific PCMRI, (2) the representative waveform (RepFlow), (3) the representative ultrasound-derived adult waveform (Kips), (4) the triangulation approach, and (5) the three virtual Doppler approaches (DopEnv, DopMean, PeakU). The forward and backward components of pressure (f and b subscripts, respectively) are defined as:

1 $${P}_{\mathrm{f}}=\frac{1}{2}\left(P-{P}_{\text{ud}}+{Z}_{\text{c}}Q\right)$$

2 $${P}_{\mathrm{b}}=\frac{1}{2}\left(P-{P}_{\text{ud}}-{Z}_{\text{c}}Q\right)$$

where ${P}_{\text{ud}}$ is the undisturbed pressure (assumed to be zero in this study), *Q* is the respective flow waveform, and ${Z}_{\text{c}}$ is the characteristic impedance [15, 35]. ${Z}_{\text{c}}$ was calculated as the slope of the early-systolic *P*–*Q* relation and has arbitrary units when *P* and *Q* are uncalibrated [36, 37].

To assess the utility of estimated flow waveforms for evaluating arterial wave reflection, we quantified reflection magnitude (RM) and timing. Reflection magnitude (RM) was calculated as

3 $$\mathrm{RM}=\frac{\Delta {P}_{\text{b}}}{\Delta {P}_{\text{f}}}$$

where $\Delta {P}_{\text{b}}$ and $\Delta {P}_{\text{f}}$ refer to the amplitude of the backward and forward components of pressure, respectively.

The return time of reflected waves (${T}_{\text{r}}$) was quantified via the ‘centroid method’ that was recently proposed and validated by our group [38]. This approach quantifies return time as the delay between the time-axis centroids of ${P}_{\text{b}}$ and ${P}_{\text{f,in}}$ waveforms, where ${P}_{\text{b}}$ is the backward component of pressure from Eq. (1) after offsetting to a minimum value of zero. ${P}_{\text{f,in}}$ is the ‘input pressure’ waveform (i.e. the pressure that would exist in the absence of wave reflection), assumed to be identical to the flow waveform, noting that ${P}_{\text{f,in}}=\mathrm{Q}{Z}_{\text{c}}$ in the absence of reflected waves, which assumes that ventricular outflow is relatively unaffected by reflected waves [38].

### Statistics

Differences in participant characteristics and wave separation indices between groups were analysed with one-way ANOVA with Bonferroni correction for multiple comparisons. Since the representative flow waveform was developed using data from Group 1, independent validation in this group was performed using a leave-one-out cross-validation approach: To evaluate the error for one individual, a representative flow waveform was generated from all data in Group 1 except data from that individual. For the next individual, another representative flow waveform was generated, again excluding that individual, and so on. A final representative flow waveform using all individuals from Group 1 was then used for analysis of Groups 2 and 3.

Differences in wave reflection indices via wave separation with subject-specific PCMRI-derived flow vs the estimation methods were assessed via repeated measures one-way ANOVA with Bonferroni correction for multiple comparisons. Statistical significance was defined as *p* < 0.05. Differences in the forward or backward waveform components for PCMRI-derived flow vs the estimation methods were quantified for cross-sectional area (${A}_{\text{f,b}}$, application of Eqs. (1) and (2) to the raw area waveforms) and pressure (${P}_{\text{f,b}}$, application of Eqs. (1) and (2) after calibration of the area waveform to measured mean and diastolic brachial blood pressure, where available) as the root mean squared error (RMSE). Note that the RMSE for the forward component and backward component was always identical, and therefore only one value is provided.

## Results

Participant characteristics for the three groups are shown in Table 1. There was no difference in age, height, weight, heart rate, left ventricular mass index or ejection fraction between Groups 1 and 2; however, Group 2 had a lower stroke volume and cardiac output. There was no difference in RM, *T*_r_, *A*_f_, *A*_b_*, P*_f_, or *P*_b_ between any of the groups, with the exception that *A*_b_ was 30% higher in Group 3 vs Group 1 (*P* = 0.004), and *P*_b_ was 22% higher in Group 3 vs Group 2 (*p* = 0.02, Table 1).

**Table 1.** Participant characteristics

	Group 1 (*n* = 45)	Group 2 (*n* = 79)	Group 3 (*n* = 29)
Age (years)	15.0 ± 2.4 (10.4–18.9)	14.9 ± 2.4 (7.8–18.7)	31.4 ± 10.5 (19.1–59.5)^ce^
Male sex, *n* (%)	28 (62)	35 (44)	13 (45)
Height (cm)	169 ± 14 (142–194)	163 ± 20 (62–200)	170 ± 12 (149–195)
Weight (kg)	63 ± 18 (33–99)	59 ± 17 (22–115)	74 ± 16 (43–112)^ae^
BSA (m^2^)	1.7 ± 0.3 (1.1–2.3)	1.6 ± 0.3 (0.9–2.5)	1.8 ± 0.2 (1.4–2.2)^e^
Heart rate (bpm)	71 ± 14 (47–102)	73 ± 14 (45–106)	71 ± 14 (52–102)
SV (mL)	92 ± 27 (39–142)	79 ± 22 (39–163)^b^	83.8 ± 15.2 (57–115)
CO (L/min)	6.4 ± 1.5 (3.5–9.0)	5.7 ± 1.5 (2.8–10.4)^a^	5.9 ± 1.2 (3.9–8.1)
EF (%)	63 ± 6 (53–76)	64 ± 6 (52–81)	63 ± 8 (47–90)
LVMI (g/m^2^)	70.3 ± 14.3 (39.0–100.0)	66.6 ± 14.2 (36.6–103.9)	66.3 ± 14.2 (34.5–100.0)
RM	0.52 ± 0.13	0.52 ± 0.09	0.56 ± 0.10
*T*_r_ (ms)	268 ± 54	274 ± 61	288 ± 47
*A*_f_ (mm^2^)	171 ± 71	190 ± 59	200 ± 53
*A*_b_ (mm^2^)	86 ± 35	98 ± 33	112 ± 35^b^
*P*_f_ (mmHg)^*^	42.5 ± 13.0	38.0 ± 14.9	44.2 ± 10.3
*P*_b_ (mmHg)^*^	21.1 ± 5.8	19.0 ± 7.5	23.2 ± 6.0^d^

^a^*P* < 0.05, ^b^*P* < 0.01, ^c^*P* < 0.001, Group 2 or 3 vs Group 1; ^d^*P* < 0.05, ^e^*P* < 0.002, Group 2 vs Group 3

^*^n = 34 (Group 1), *n* = 70 (Group 2), n = 25 (Group 3) due to missing blood pressure data

Data presented as mean ± SD (range) or number (%)

**Fig. 2:**
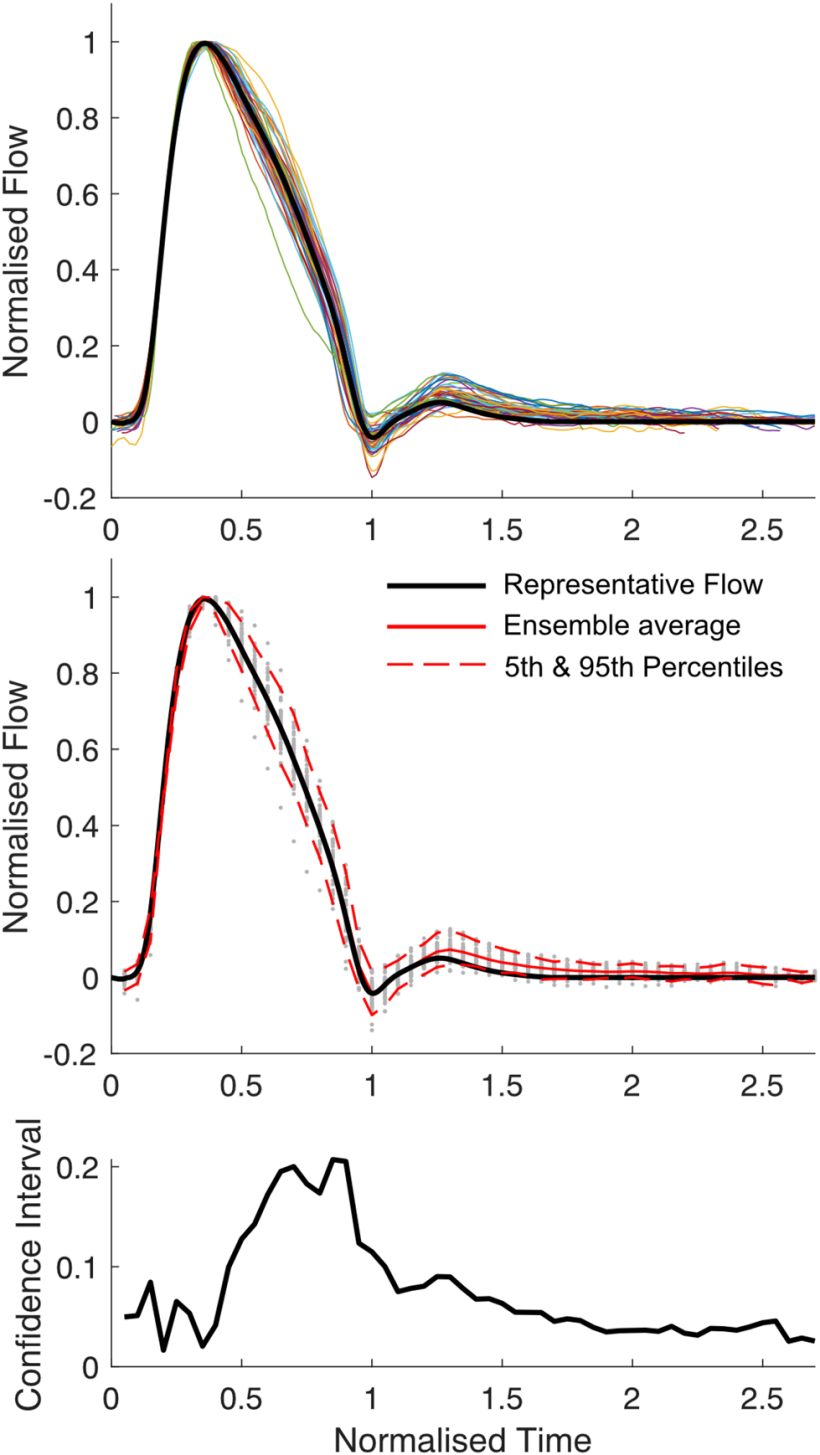
Average flow waveform for patients with normal cardiac function and aorta. (**Top**) Individual flow waveforms (coloured lines) and population-derived waveform (black line). (**Middle**) Population-derived representative waveform (black line), ensemble average flow waveform (solid red line, visible only during diastole, otherwise identical to black line), 5th and 95th percentiles (dashed red lines), with individual data points (grey dots) plotted at normalized time intervals of 0.05. (**Bottom**) Width of 5-95% confidence interval.

The individual, ensemble-averaged and representative flow (RepFlow) waveforms for Group 1 are displayed in Fig. 2. Raw data for the RepFlow waveform are provided in a Data Supplement. It can be seen that the normalized waveforms were highly consistent, with almost no variation during the systolic upstroke, and modest variation during the systolic downstroke. A small difference between the ensemble-averaged and RepFlow waveform can be seen during diastole due to application of the ramp function enforcing zero flow by late-diastole (Fig. 2, middle panel, solid red vs black lines). The 5-95% confidence interval (distance between dashed red lines in the middle panel) reached up to 0.2 normalized units (bottom panel), suggesting that the flow waveform deviated from the representative waveform by a maximum of only ~ 10% of peak flow. At 75% of ejection time (approximate time where the maximum deviation occurred, based on Fig. 2, bottom panel), linear regression revealed that this deviation was associated with *P*_f_ at this time (normalized to Δ*P*_f_, *R* = 0.48, *p* < 0.001), but not *P*_b_ (*p* = 0.9), *RM* (*p* = 0.3) or *T*_r_ indexed to ejection time (*p* = 0.8) in Group 1. By contrast, in Groups 2 and 3, both *P*_f_ and *P*_b_ (normalized to Δ*P*_f_) at 75% of ejection time were associated with the deviation (*R = *0.66 and 0.36, respectively, *p* < 0.002, Group 2; *R = *0.42 and 0.37, *p* < 0.05, Group 3), but *RM* and *T*_r_ were not.

**Fig. 3:**
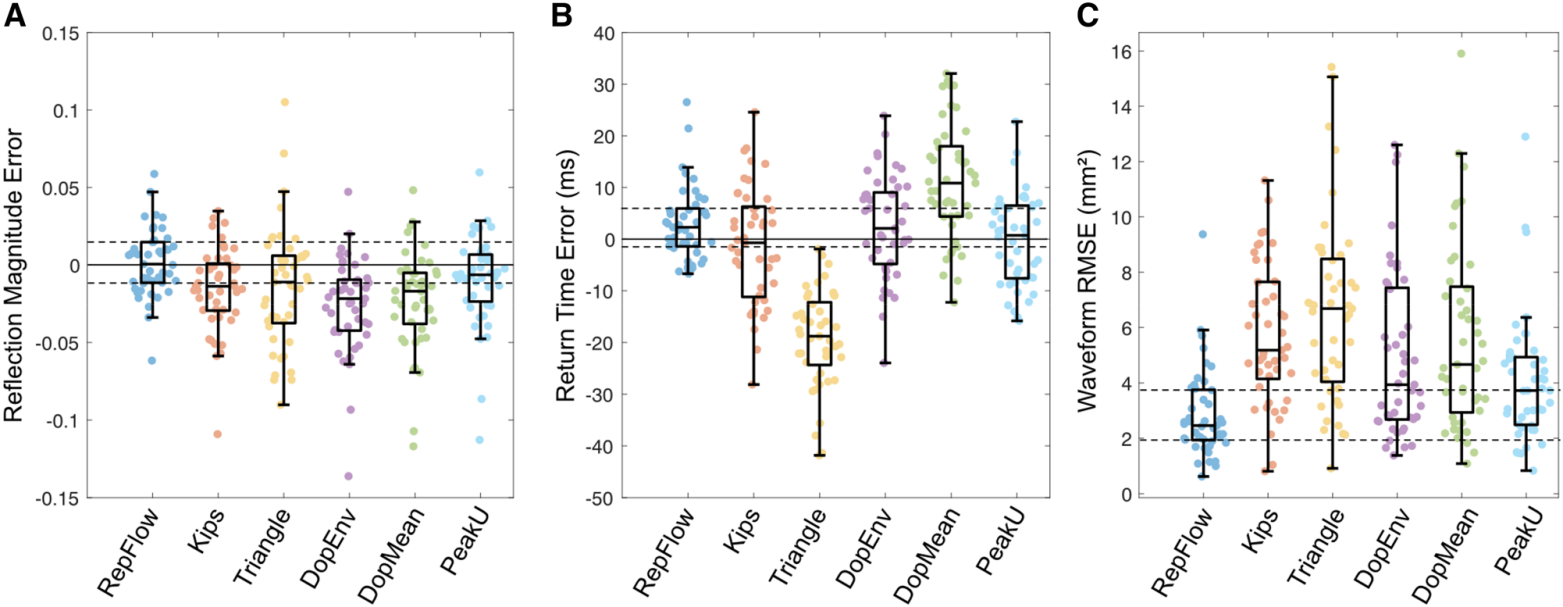
Errors in calculated **A** reflection magnitude, **B** reflected wave return time, and **C** root mean square error for forward/backward waveform components. Data are from Group 1 (children/adolescents with normal left ventricle and aorta), obtained with a representative PCMRI-derived flow waveform (RepFlow); representative Doppler-derived adult flow waveform from Kips et al. [28] with a triangular flow waveform; with a virtual Doppler ultrasound applied to the spectral envelope (DopEnv) or mean velocity (DopMean) within a realistic sample volume; or with peak velocity in the whole lumen (PeakU). Individual errors were calculated via leave-one-out cross-validation. The solid horizontal lines in panels A and B correspond to the zero error line; the dashed horizontal lines indicate the interquartile range for RepFlow as a reference.

Results of wave separation are shown in Table 2, including raw values, percentage errors and percentage absolute errors for *RM* and *T*_r_; raw errors are shown in Figs. 3, 6 and 7. There were no significant differences between any groups for *RM* (*p* = 0.91, Group 1 vs Group 2, *p* = 0.13, Group 1 vs Group 3; *p* = 0.33, Group 2 vs Group 3) or *T*_r_ (all *p* > 0.4) derived from PCMRI.

**Table 2.** Reflection magnitude and accuracy of wave separation for different flow waveforms

	PCMRI	RepFlow	Kips	Triangle	DopEnv	DopMean	PeakU
Group 1 (*n* = 45)							
RM (–)	0.52 ± 0.13	0.52 ± 0.13	0.51 ± 0.12^c^	0.51 ± 0.12^c^	0.50 ± 0.13^a^	0.50 ± 0.12^a^	0.51 ± 0.13
RM Error (%)	–	0.4 ± 4.2	− 2.6 ± 4.8^e^	− 2.3 ± 7.9^f^	− 4.9 ± 5.9^d^	− 4.2 ± 5.8^d^	− 1.8 ± 5.5
|RM Error| (%)	–	3.3 ± 2.5	4.3 ± 3.3	6.1 ± 5.4^e^	5.8 ± 4.9^e^	5.6 ± 4.4^f^	4.0 ± 4.1
* T*_r_ (ms)	268 ± 54	271 ± 55	267 ± 54	249 ± 51^a^	271 ± 56	279 ± 58^a^	268 ± 58
* T*_r_ Error (%)	–	1.1 ± 2.7	− 0.4 ± 4.5	− 7.2 ± 3.5^d^	0.9 ± 3.8	4.0 ± 4.2^e^	− 0.4 ± 3.5
|*T*_r_ Error| (%)	–	2.1 ± 2.0	3.6 ± 2.7^f^	7.2 ± 3.5^d^	3.1 ± 2.2	4.8 ± 3.3^d^	2.9 ± 2.0
RMSE (*A*_f,b_, mm^2^)	–	2.8 ± 1.6	5.7 ± 2.5^d^	6.6 ± 3.3^d^	4.9 ± 3.0^d^	5.6 ± 3.3^d^	4.1 ± 2.3^e^
RMSE (*P*_f,b_*,* mmHg)^g^	–	0.8 ± 0.5	1.5 ± 0.6^d^	1.6 ± 0.7^d^	1.2 ± 0.7^d^	1.5 ± 0.9^d^	1.1 ± 0.6
Group 2 (*n = 79*)							
RM (–)	0.52 ± 0.09	0.50 ± 0.09	0.48 ± 0.08^a^	0.47 ± 0.08^a^	0.48 ± 0.08^a^	0.52 ± 0.10	0.49 ± 0.09^a^
RM Error (%)	–	− 2.9 ± 6.1	− 7.0 ± 6.6^e^	− 8.8 ± 9.4^d^	− 6.5 ± 9.6^e^	1.4 ± 10.6^e^	− 5.1 ± 9.6
|RM Error| (%)	–	5.1 ± 4.5	7.8 ± 5.7^e^	10.9 ± 6.9^d^	9.1 ± 7.1^d^	7.8 ± 7.2^e^	8.2 ± 7.1^e^
* T*_r_ (ms)	274 ± 61	278 ± 61	274 ± 64	251 ± 58^a^	282 ± 63^a^	291 ± 65^a^	273 ± 60
* T*_r_ Error (%)	–	1.7 ± 3.3	− 0.0 ± 5.5	− 8.6 ± 3.6^d^	3.1 ± 6.0	6.5 ± 6.6^d^	− 0.2 ± 5.8^f^
|*T*_r_ Error| (%)	–	3.1 ± 2.1	4.1 ± 3.6	8.6 ± 3.6^d^	5.3 ± 4.2^d^	7.7 ± 5.1^d^	4.3 ± 3.8
RMSE (*A*_f,b_, mm^2^)	–	5.1 ± 3.2	8.5 ± 4.1^d^	8.4 ± 3.3^d^	8.6 ± 5.8^d^	10.3 ± 7.5^d^	7.0 ± 4.7^e^
RMSE (*P*_f,b_*,* mmHg)^g^	–	1.0 ± 0.6	1.7 ± 0.9^d^	1.7 ± 0.8^d^	1.7 ± 1.2^d^	2.1 ± 1.5^d^	1.4 ± 1.0^e^
Group 3 (*n = *29)							
RM (–)	0.56 ± 0.10	0.53 ± 0.09^c^	0.51 ± 0.09^a^	0.49 ± 0.08^a^	0.48 ± 0.08^a^	0.52 ± 0.09^a^	0.48 ± 0.07^a^
RM Error (%)	–	− 4.5 ± 6.5	− 8.3 ± 6.2	− 11.6 ± 10.4^d^	− 13.0 ± 8.6^d^	− 5.7 ± 6.7	− 13.8 ± 8.2^d^
|RM Error| (%)	–	6.2 ± 4.8	9.1 ± 4.9	13.4 ± 7.8^d^	13.9 ± 7.0^d^	6.7 ± 5.7	14.1 ± 7.7^d^
* T*_r_ (ms)	288 ± 47	286 ± 49	284 ± 49	265 ± 44^a^	294 ± 56	305 ± 54^a^	286 ± 51
* T*_r_ Error (%)	–	− 0.6 ± 3.5	− 1.2 ± 4.9	− 7.8 ± 3.1^d^	1.9 ± 6.6	6.0 ± 6.2^d^	− 0.7 ± 4.5
|*T*_r_ Error| (%)	–	2.9 ± 2.1	3.8 ± 3.3	7.8 ± 3.1^d^	5.3 ± 4.3^e^	7.2 ± 4.7^d^	3.5 ± 2.8
RMSE (*A*_f,b_, mm^2^)	–	5.5 ± 2.9	8.2 ± 4.4^f^	9.0 ± 3.0^e^	10.8 ± 5.0^d^	12.9 ± 8.4^d^	10.5 ± 7.2^d^
RMSE (*P*_f,b_*,* mmHg)^g^	–	1.3 ± 0.7	1.6 ± 0.6	2.1 ± 0.8^e^	2.3 ± 1.3^d^	2.8 ± 2.1^d^	2.0 ± 1.0^e^

^a^*p* ≤ 0.001, ^b^*p* ≤ 0.01, ^c^*p* < 0.05, compared with PCMRI; ^d^*p* ≤ 0.001, ^e^*p* ≤ 0.01, ^f^*p* < 0.05, compared with RepFlow; ^g^*n* = 34 (Group 1), *n* = 70 (Group 2), *n* = 25 (Group 3) due to missing blood pressure data. PCMRI, subject-specific flow waveform from phase contrast magnetic resonance imaging; RepFlow, representative flow waveform derived from PCMRI in Group 1 in the present study; Kips, representative flow waveform from ultrasound in adults reported by Kips et al. [28]; Triangle, triangular flow waveform as in Westerhof et al. [27] with peak at 25% of systolic duration; DopEnv, using flow waveform obtained by tracing the envelope of the virtual Doppler ultrasound; DopMean, using flow waveform obtained as the intensity-weighted spectral average from the virtual Doppler ultrasound; PeakU, flow waveform obtained as the instantaneous peak velocity within the lumen. Vertical bars (as in |RM Error|) refer to absolute values. *RM* reflection magnitude, *RMSE* root mean square error for forward or backward waveforms of area (*A*_f,b_) and pressure (*P*_f,b_), *T*_r_ return time of the reflected wave.

In Group 1, using the leave-one-out cross-validation approach, there was no difference between PCMRI and RepFlow for *RM*, whereas all other approaches underestimated *RM* (Table 2) and absolute RM errors were 1.5–3 times greater than those from RepFlow (except for PeakU). RepFlow, Kips, DopEnv, and PeakU did not differ statistically from PCMRI for *T*_r_, whereas Triangle and DopMean underestimated and overestimated *T*_r_, respectively. However, absolute errors for *T*_r_ were lowest for RepFlow (although the comparison with DopEnv and PeakU did not reach significance; Table 2 and Supplemental Figure 2). When comparing forward/backward component waveforms against PCMRI-derived wave separation, RepFlow also exhibited lower waveform RMSE for both area (Fig. 3C, Table 2) and pressure (Table 2), compared with the other methods.

**Fig. 4:**
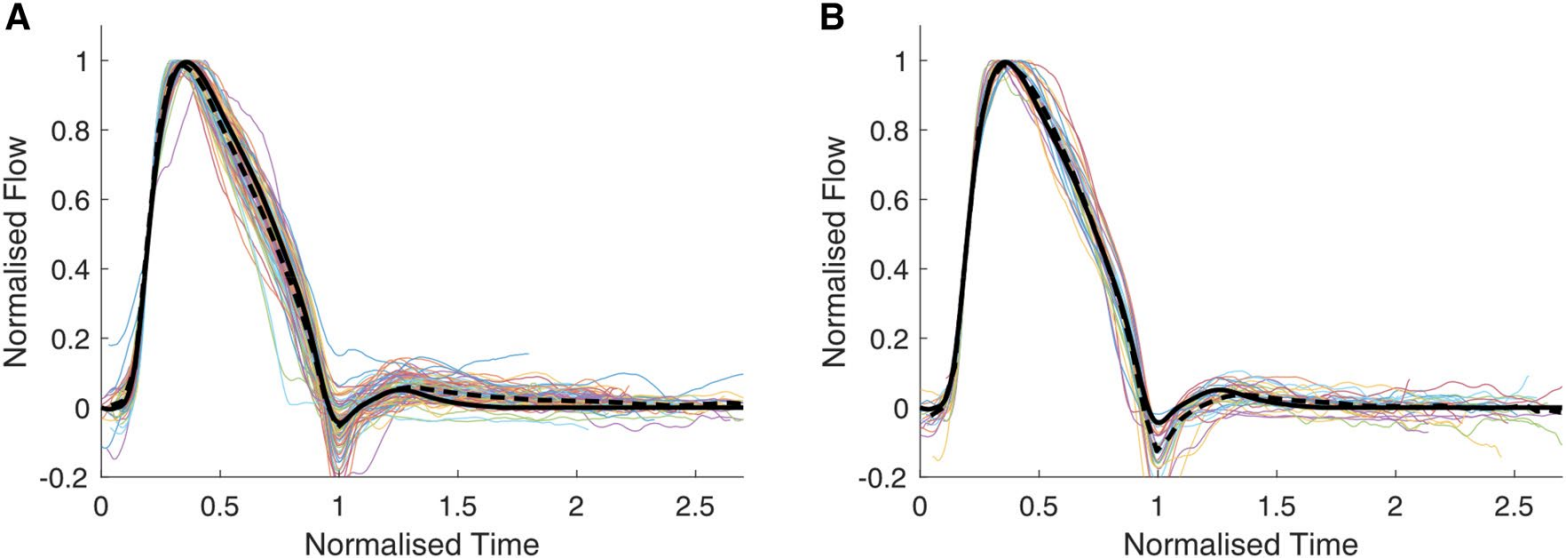
Individual flow waveforms from PCMRI (coloured lines), the ensemble-averaged waveform (dashed black line) for **A** children and adolescents with childhood heart disease (Group 2); and **B** adult congenital heart disease patients (Group 3). The representative flow (RepFlow) waveform developed from Group 1 data is also shown for reference (solid black line)

**Fig. 5:**
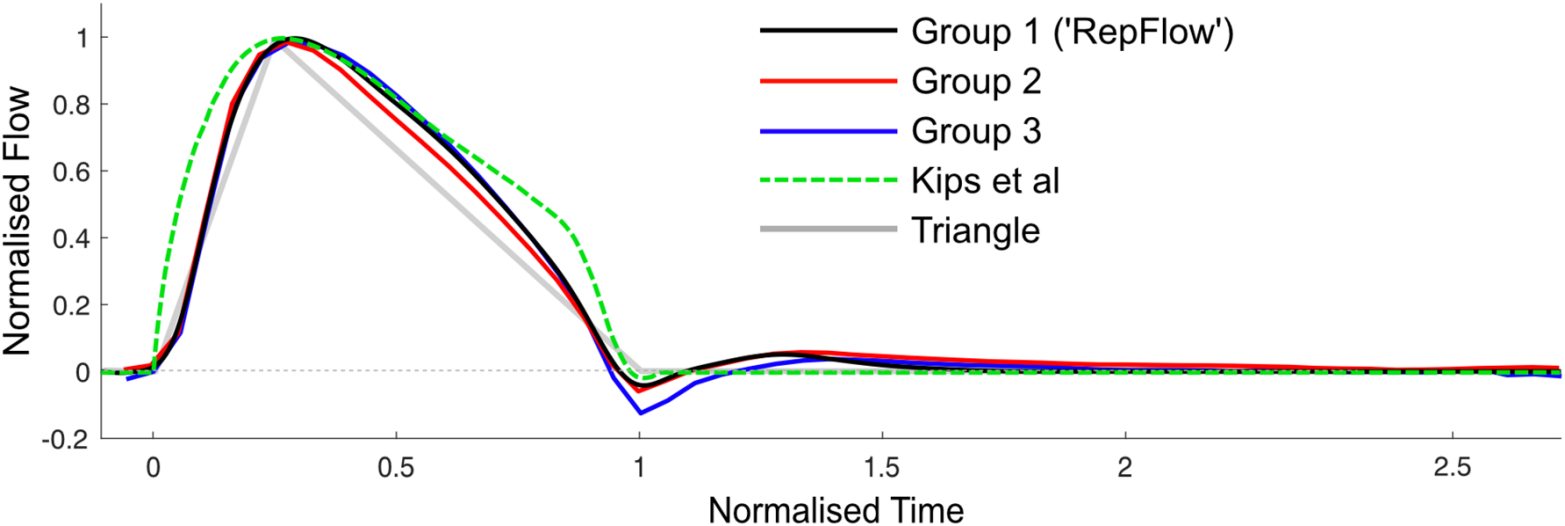
Comparison of averaged flow waveforms from Group 1 (‘RepFlow’), Group 2 and Group 3 derived from PCMRI, as well as the representative flow waveform derived from Doppler ultrasound in adults by Kips et al. [28]

Figure 4 displays the individual flow waveforms from PCMRI for Groups 2 and 3. The representative flow waveform (developed using data from Group 1) closely approximated the ensemble-averaged waveform for both Groups 2 and 3. As in Group 1, most of the variability in the waveform occurred during flow deceleration. Figure 5 compares RepFlow (average waveform for Group 1) with the average waveforms from Groups 2 and 3, as well as Kips and Triangular waveforms.

**Fig. 6:**
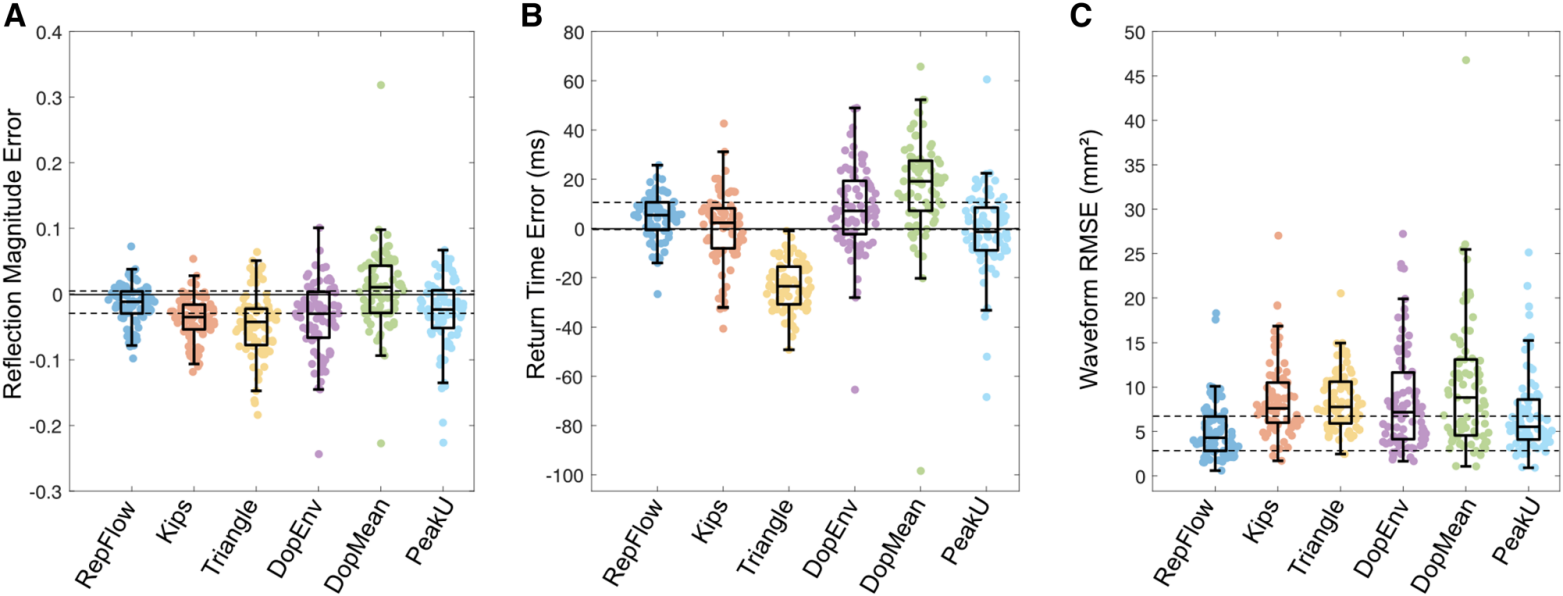
Errors in calculated **A** reflection magnitude, **B** reflected wave return time and **C** root mean square error for forward/backward waveform components in Group 2 (childhood heart disease). Data are presented as in Fig. 3.

**Fig. 7:**
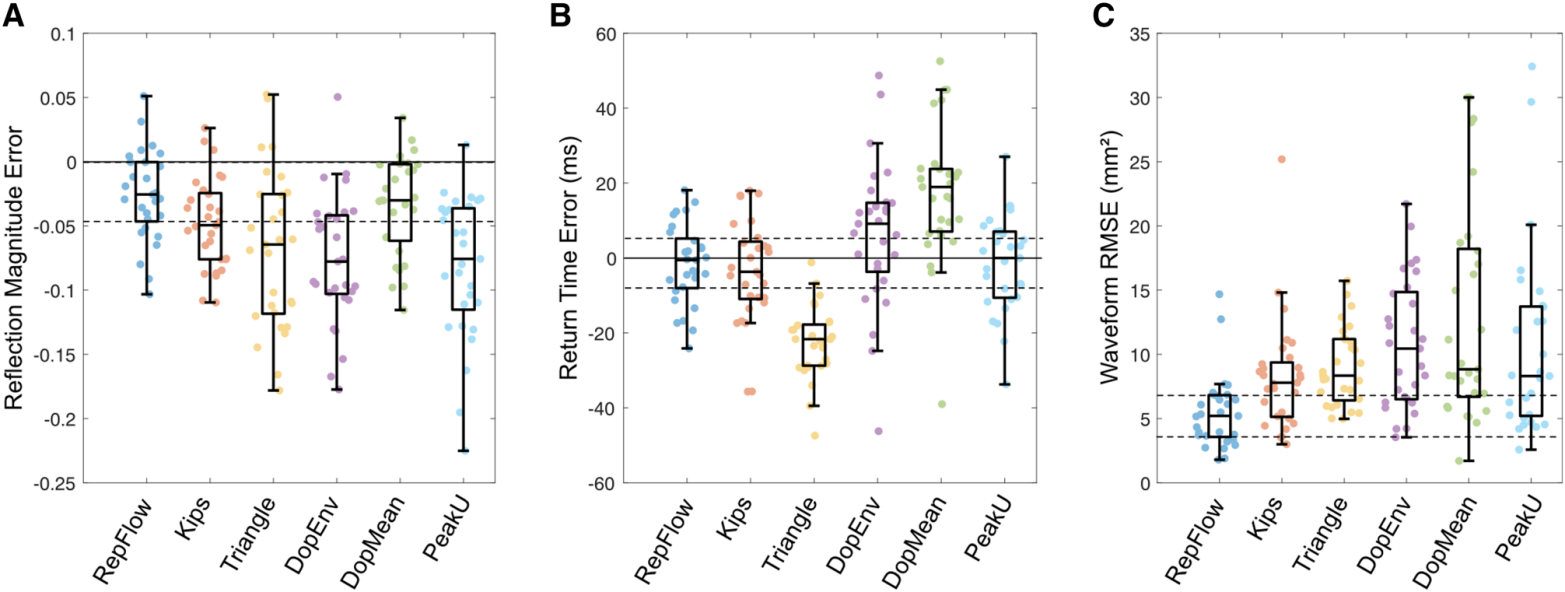
Errors in calculated **A** reflection magnitude, **B** reflected wave return time and **C** root mean square error for forward/backward waveform components for Group 3 (adult congenital heart disease patients). Data are presented as in Fig. 3.

Errors in calculated *RM*, *T*_r_ and component waveforms compared with PCMRI-derived quantities for Groups 2 and 3 are shown in Table 2 and Figs. 6 and 7. RepFlow did not differ from PCMRI for *RM* in Group 2, *T*_r_ in both groups, and underestimated *RM* in Group 3 by less than 5% on average. *RM* and *T*_r_ derived from the other methods yielded errors that were typically 1.5–3 times greater than RepFlow (Table 2 and Figure S2 in the Supplemental Material). RMSE errors for the waveform components when using RepFlow were significantly less than all other techniques.

## Discussion

In this study, the gold-standard PCMRI technique for measuring volumetric flow in the ascending aorta demonstrated that time- and amplitude-normalized flow waveforms were highly consistent in children and adolescents. Moreover, the representative flow (RepFlow) generated via averaging in Group 1 (children and adolescents without a history of CHD) was almost identical to the averaged flow waveforms in children/adolescents and adults with a history of CHD (Groups 2 and 3). Because of this consistency, wave separation performed with RepFlow yielded errors that were generally less than 10% for indices of wave reflection (*RM* and *T*_r_) in all groups, along with similar forward/backward component waveforms. RepFlow outperformed the triangular flow method [27], a representative adult flow waveform based on ultrasound data [28], and was even superior to the predicted best-case patient-specific measured flow that would be obtained via Doppler ultrasound.

To our knowledge, this is the first study to reveal the high level of consistency in ascending aortic flow waveform morphology in children and adolescents with and without a history of CHD, spanning a wide range of ages (7–18 years), heart rates (45–102 bpm), and cardiac outputs (2.8–10.4 L/min, see Table 1), despite the abnormalities in ventricular and/or arterial properties that may be present in the CHD group [6, 8, 11, 39, 40]. Moreover, almost identical flow waveforms were also found in adults with a history of CHD. These results suggest that the natural LV outflow pattern under resting conditions is relatively fixed, particularly during the flow upstroke, where the normalized waveforms varied by only ± 2.5% of peak flow (based on the confidence interval of ~ 0.05 normalized units during this time, Fig. 2).

Greater variation (although still only ± 10%) was seen during the flow downstroke, which, based on prior work in adults [41], might be expected to indicate a variable flow-reducing effect of mid-to-late systolic wave reflection. However, in Group 1, we found no association of this variation with reflection indices, but a strong association with the forward wave, implying a predominant influence of LV or proximal aortic properties. By contrast, in both younger and older CHD groups, the deviation of mid-to-late systolic flow from RepFlow was associated with forward *and* backward pressure components, indicating some impact of wave reflection on LV outflow. This effect may be subtle, however, as no statistically significant differences in reflection magnitude or return time were detected between groups.

Whilst PCMRI is considered the gold standard for non-invasive measurement of volumetric blood flow in large arteries [42], Doppler ultrasound is more commonly used in wave analysis studies [28, 31, 32, 43–47], likely due to its greater accessibility and lower cost. Our investigation of a virtual Doppler ultrasound based on PCMRI data represented several best-case scenarios, where envelope (*DopEnv*) and mean blood velocities (*DopMean*) within a typical sample volume were obtained without the errors that may arise from factors such as limited acoustic window, angle dependence, operator dependence, and physics-related limitations of ultrasound [33, 48]. Moreover, *PeakU* represented the envelope that would be obtained with an ideal sample volume that precisely covers the entire lumen at every time point. It was therefore perhaps surprising that none of these best-case subject-specific approaches outperformed the patient-generic RepFlow approach for wave separation analysis. As exemplified in Fig. 1, and similar to published results for the carotid artery [33], differences between the true mean velocity (and hence flow) waveforms and *DopEnv*, *DopMean*, and *PeakU* waveforms arose due to a combination of velocity profile skewing (e.g. due to curvature in the flow path) and limited coverage of the lumen by the sample volume. These differences translated into errors in calculated reflection magnitude and return time.

The limitations of Doppler ultrasound may be relevant when considering the results obtained from the representative flow waveform used by Kips et al. [28] Whilst it could be argued that the better results in children/adolescents (Groups 1 and 2) using RepFlow compared with the Kips waveform arose because the latter was obtained from data in adults, similar results were also found in Group 3 (ages 19 to 59 years, albeit with a mean age of 31 vs 45 in Kips et al. [28]). Whether the differences in waveform shape (such as more convex flow upstroke and more prominent shoulder on the downstroke, see Supplemental Figure 1) arise from age differences and/or limitations of the ultrasound approach requires further study. However, our results suggest that the broader generalizability of RepFlow in adults (e.g. in the non-CHD population and in older individuals) is worthy of investigation.

The triangulation approach proposed by Westerhof et al. [27] is implemented in at least one leading commercial device [49] and has been widely used in clinical and population-based studies in adults [22, 50–52], along with one study in children and adolescents [53]. Our results indicate that the triangulation method tends to underestimate reflection magnitude and return time, with a wide spread of errors. The smooth curvature of the actual flow waveform likely underlies these errors, as adjusting the time of the triangle apex did not appear to improve results.

Armstrong et al. [31] recently published an investigation of pressure-only wave separation in adolescents (age 14–19 years). The present work is distinct in several respects: (1) the cohort included younger patients (down to 7 years in Group 2), (2) both reflection magnitude and return time were investigated, (3) the reference method was PCMRI rather than ultrasound, and (4) our cohort included children, adolescents, and adults with a history of childhood heart disease.

Our results should be interpreted with respect to several methodological considerations and limitations. First, we used cross-sectional area waveforms derived from PCMRI as a surrogate of pressure waveforms; although this had the benefit of being centrally obtained (not dependent on transfer functions), the waveforms had a relatively low temporal resolution (128 frames per cycle) and may differ from actual pressure waveforms due to the wall’s non-linear viscoelasticity. However, data from a nearby central elastic artery (carotid) suggest this effect is likely to be minor [54, 55], and values of reflection magnitude were similar to those in previous reports that employed pressure waveforms [27, 28, 30, 32, 56]. In addition, unpublished data from our group show similar values for return time when obtained via carotid tonometry and the centroid method [38]. Second, since this study used retrospective data, Doppler ultrasound measurements of ascending aortic flow were not available for comparison with the virtual ultrasound; however, our aim was to investigate the best-case scenario and actual measurements would involve additional sources of error. Third, our virtual ultrasound results were obtained in the ascending aorta, whereas it is common to acquire data in the LV outflow tract [28, 31, 32]; however, velocity profile skewing has also been shown to be present in the outflow tract [57, 58]. Fourth, a number of other pressure-only wave separation methods have been described that were not investigated herein, such as those based on the model-based (ARCSolver) approach described by Hametner et al. [29] and the subject-specific synthesized waveform described by Shenouda et al. [30] which involve codes that are not publicly available. Interestingly, the RMSE of the separated pressure waveforms for RepFlow was essentially identical to that previously reported with the subject-specific ARCSolver approach in adults (~ 0.8 mmHg) [29] as were absolute differences in reflection magnitude (3% ± 2%) in Group 1 of the present study and in young adults in Shenouda et al. [30] Fifth, our cohort only included patients with normal ejection fraction (> 50%). Ventricular outflow in patients with low ejection fraction may be more affected by wave reflection, leading to greater variability in flow waveform shape, and potentially lower accuracy of wave separation with RepFlow [32, 59]. Finally, since our data did not include young children or infants, the applicability of RepFlow in this age group requires further study.

In conclusion, the high degree of uniformity in ascending aortic flow waveform morphology in children and adolescents (with or without a history of CHD) enabled wave separation analysis to be performed with a high degree of accuracy using only a pressure (or area) waveform and a representative flow waveform derived from gold-standard PCMRI. This RepFlow method outperformed the triangulation method, a representative adult waveform derived from Doppler ultrasound, and even best-case (virtual) subject-specific Doppler ultrasound. RepFlow also outperformed other techniques in a group of adults with a history of CHD, implying that wave separation with RepFlow may be applicable in a wide variety of settings.

### Supplementary Information

Below is the link to the electronic supplementary material.

Supplementary file1 (PDF 3268 kb)
