# Supplementary material for: Superiority of a Representative MRI Flow Waveform over Doppler Ultrasound for Aortic Wave Reflection Assessment in Children and Adolescents With/Without a History of Heart Disease
Source: Ann Biomed Eng. 2023 Aug 10;51(12):2772–84. doi: 10.1007/s10439-023-03339-2 (PMC10632254; doi:10.1007/s10439-023-03339-2)
Supplement: Supplementary file 1 — Supplementary file1 (PDF 3268 kb) [file 10439_2023_3339_MOESM1_ESM.pdf]

Supplementary Material

Superiority of a representative MRI flow waveform over Doppler ultrasound for aortic wave reflection assessment in children and adolescents with/without a history of heart disease

Jonathan P. Mynard,[a,b,c] Remi Kowalski,[a,b,d] Hilary A. Harrington,[a] Avinash Kondiboyina,[a,b] Joseph J. Smolich,[a,b] Michael M.H. Cheung[a,b,d]

[a] Heart Research, Murdoch Children's Research Institute, Parkville, VIC Australia
[b] Department of Paediatrics, University of Melbourne, Parkville VIC, Australia
[c] Department of Biomedical Engineering, University of Melbourne, Parkville VIC, Australia
[d] Department of Cardiology, Royal Children's Hospital, Parkville VIC, Australia

Patient Characteristics

Patients in Group 2 had a history of anomalous left coronary artery from the pulmonary artery and mitral regurgitation (n=1), aortic stenosis with dilated ascending aorta (1), bicuspid aortic valve (4), coarctation of the aorta (19), interrupted aortic arch (1), Loeys-Dietz syndrome (3), Marfan syndrome (11), partial anomalous pulmonary venous drainage (4), tetralogy of Fallot (9), transposition of the great arteries (19) and Turner's syndrome (15), noting that some individuals had multiple diagnoses.

Patients in Group 3 had a history of bicuspid aortic valve (n=1), coarctation of the aorta (5), pulmonary regurgitation (8), tetralogy of Fallot (19), transposition of the great arteries (5), truncus arteriosus (1), and Turner's syndrome (2), which also included subjects with multiple diagnoses.

Image Segmentation

After initial cropping to a region of interest around the ascending aorta (Figure **S1**A) and approximate localization of the aortic centre point via a manually drawn circle around the aortic perimeter, a series of eight M-lines passing through the centre point were created (for example, dashed line in Figure **S1**B). An M-mode image was then generated by mapping pixel intensities along a given M-line over time (using Matlab's *improfile* function with bicubic interpolation, Figure **S1**C). To account for variations in contrast over time arising from variations in flow magnitude during the cardiac cycle, intensities in each column were scaled to achieve an approximately uniform mean intensity within the lumen (Figure **S1**C). Upper and lower contours were then traced using a semi-automated intensity-tracking technique, whereby clicking and dragging the mouse left or right (i.e. along the time axis) propagated the contour. Moving the mouse up or down adjusted the intensity value, $\mathrm{I}(t_0, y)$, that was being

locked on to, where t_0 is the time co-ordinate where the mouse was first clicked, and y is the co-ordinate along the M-line at the current mouse position. The contour was propagated from one column to the next (i.e. from t_k to t_{k+1}, where $k = 0 \ldots n-1$) by i) calculating the absolute difference between M-line intensities and the reference intensity value, $D(t_k, y) = abs[I(t_k, y) - I(t_0, y_0)]$, and ii) finding the nearest local minima in D to the identified contour point in the previous column. Additional manual adjustments were made as required.

The distance between upper and lower contours provided a diameter waveform for each M-line, and diameter waveforms for all M-lines were checked for consistency (Figure **S1**D). Finally, the identified contour points were mapped back to the original 2D image slices (small circles in Figure **S1**B), and an ellipsoid was fitted to these, from which cross-sectional area at each time point was calculated (Figure **S1**E). Volumetric flow was obtained by integrating the velocities within the lumen. Given that the resulting waveform is a time-dependent variable, we have found that our approach (creating contours in space-time axes rather than segmenting each frame independently in 2D space) minimizes artefactual fluctuations in the waveform.

Figure S1. Segmentation procedure for the phase contrast MRI of the ascending aorta: A) Region of interest selection; B) cross-sectional view (2D in space); C) M-mode view (space-time view for the M-line shown in B); D) diameter waveforms for each M-line; E) final area waveform.

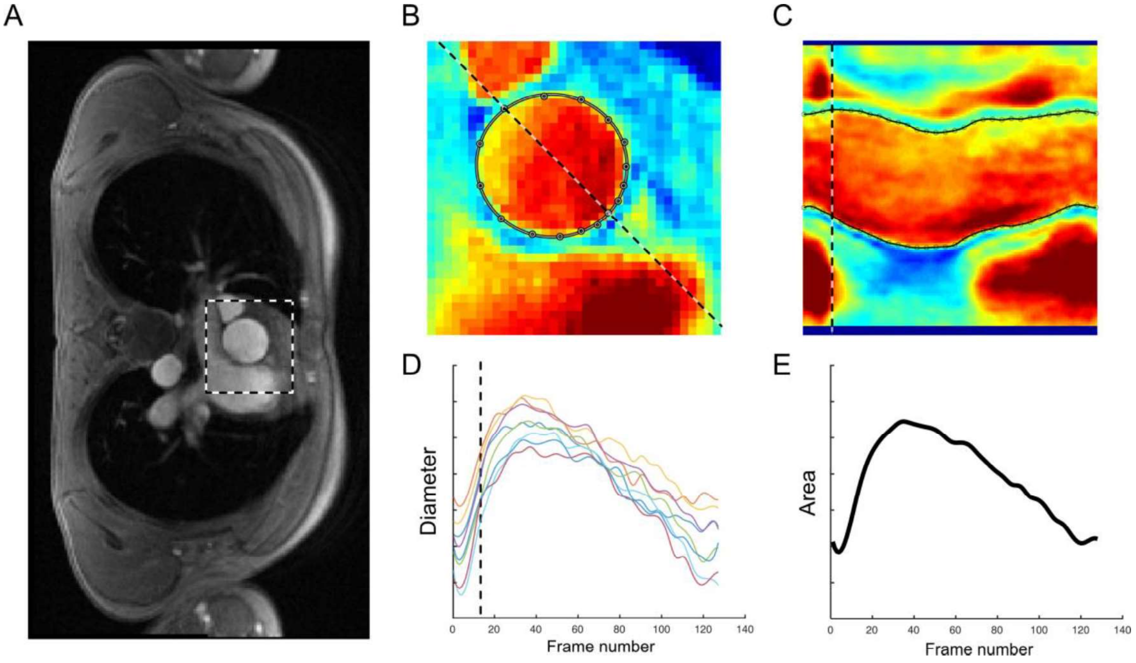

Figure S2. Percentage absolute errors for reflection magnitude (top panels) and return time (bottom panels) for the three study groups. Dashed lines indicate 5% and 10% errors for reference.

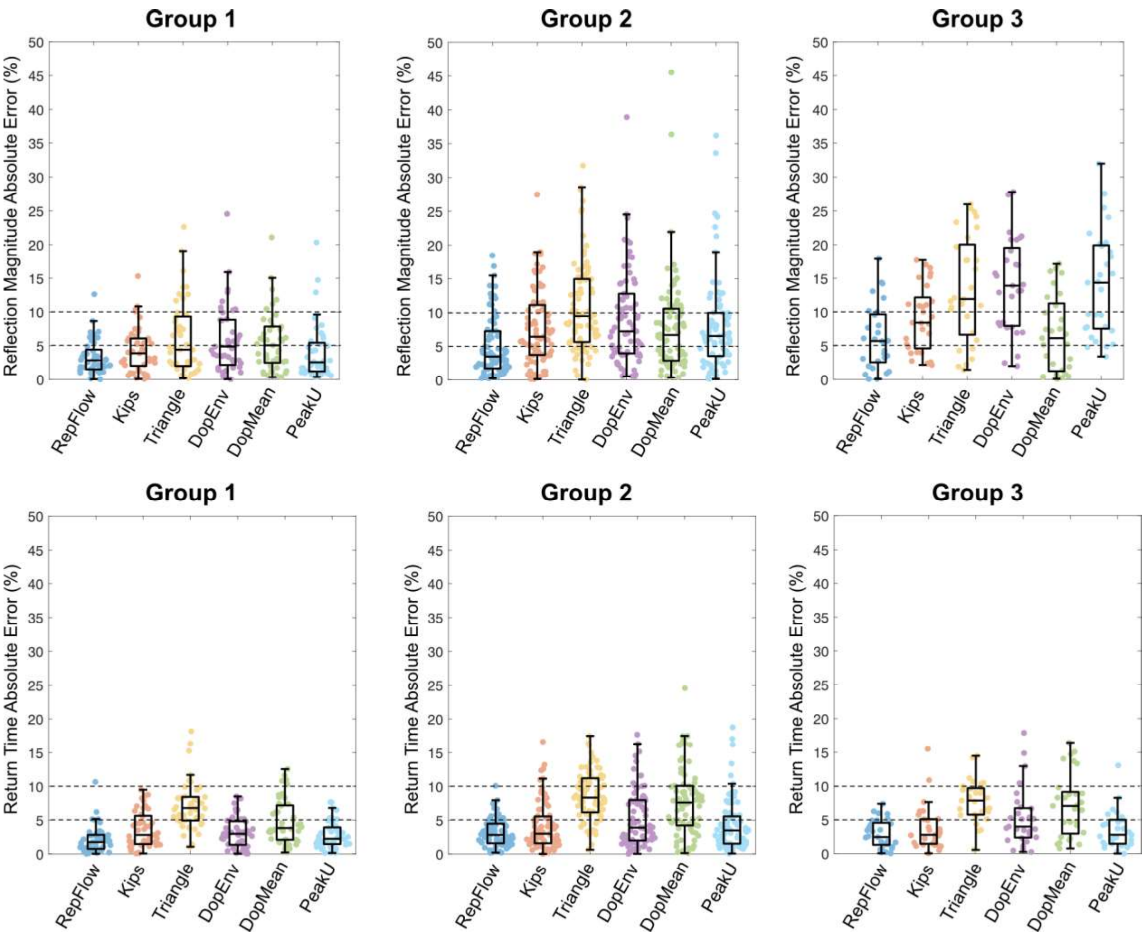

Time (arbitary units)	Normalised Flow
0	0
0.003333333	0
0.006666667	0
0.01	0
0.013333333	0
0.016666667	0
0.02	0
0.023333333	0
0.026666667	0
0.03	0
0.033333333	0
0.036666667	0
0.04	0
0.043333333	0
0.046666667	0
0.05	0
0.053333333	0
0.056666667	0
0.06	0
0.063333333	0
0.066666667	0
0.07	0
0.073333333	0
0.076666667	0
0.08	0
0.083333333	0
0.086666667	0
0.09	0
0.093333333	0
0.096666667	0
0.1	0
0.103333333	0
0.106666667	0
0.11	0
0.113333333	0
0.116666667	0
0.12	0
0.123333333	0
0.126666667	0
0.13	0
0.133333333	0
0.136666667	0

0.14	0
0.143333333	0
0.146666667	0
0.15	0
0.153333333	0
0.156666667	0
0.16	0
0.163333333	0
0.166666667	0
0.17	0
0.173333333	0
0.176666667	0
0.18	0
0.183333333	0
0.186666667	0
0.19	0
0.193333333	0
0.196666667	0
0.2	0
0.203333333	0
0.206666667	0
0.21	0
0.213333333	0
0.216666667	0
0.22	0
0.223333333	0
0.226666667	0
0.23	0
0.233333333	0
0.236666667	0
0.24	0
0.243333333	0
0.246666667	0
0.25	0
0.253333333	0
0.256666667	0
0.26	0
0.263333333	0
0.266666667	0
0.27	0
0.273333333	0
0.276666667	0
0.28	0

0.283333333	0
0.286666667	0
0.29	0
0.293333333	0
0.296666667	0
0.3	0
0.303333333	0
0.306666667	0
0.31	0
0.313333333	0
0.316666667	0
0.32	0
0.323333333	0
0.326666667	0
0.33	0
0.333333333	0
0.336666667	-0.001065633
0.34	-0.002045837
0.343333333	-0.002908048
0.346666667	-0.003619702
0.35	-0.004148234
0.353333333	-0.004461079
0.356666667	-0.004525673
0.36	-0.004309452
0.363333333	-0.00377985
0.366666667	-0.002904303
0.37	-0.001650247
0.373333333	1.49E-05
0.376666667	0.000700534
0.38	0.001384032
0.383333333	0.002424592
0.386666667	0.003808713
0.39	0.006247572
0.393333333	0.009417809
0.396666667	0.013520527
0.4	0.018684972
0.403333333	0.025043838
0.406666667	0.032925855
0.41	0.042574262
0.413333333	0.054133561
0.416666667	0.06786613
0.42	0.083944657
0.423333333	0.102434133

0.426666667	0.123343277
0.43	0.146723547
0.433333333	0.172458913
0.436666667	0.200325843
0.44	0.230174138
0.443333333	0.261796292
0.446666667	0.294898067
0.45	0.329140648
0.453333333	0.36419938
0.456666667	0.399666195
0.46	0.435220943
0.463333333	0.470542951
0.466666667	0.50535598
0.47	0.539430695
0.473333333	0.572547058
0.476666667	0.604552109
0.48	0.635350913
0.483333333	0.664837393
0.486666667	0.692905423
0.49	0.719540156
0.493333333	0.744755663
0.496666667	0.768556664
0.5	0.790967599
0.503333333	0.812003186
0.506666667	0.831676468
0.51	0.850051846
0.513333333	0.867162684
0.516666667	0.883009541
0.52	0.897650537
0.523333333	0.911151451
0.526666667	0.923522245
0.53	0.934762486
0.533333333	0.944884044
0.536666667	0.953919774
0.54	0.961918032
0.543333333	0.968957936
0.546666667	0.975112736
0.55	0.980393821
0.553333333	0.984837761
0.556666667	0.988483927
0.56	0.99137256
0.563333333	0.993495955
0.566666667	0.994892982

0.57	0.995633081
0.573333333	0.995753127
0.576666667	0.995283819
0.58	0.994254446
0.583333333	0.99268897
0.586666667	0.990619616
0.59	0.988120046
0.593333333	0.985222589
0.596666667	0.981936896
0.6	0.978301481
0.603333333	0.974363607
0.606666667	0.970141021
0.61	0.965629945
0.613333333	0.960850231
0.616666667	0.955825754
0.62	0.950590272
0.623333333	0.945160303
0.626666667	0.939556763
0.63	0.933773831
0.633333333	0.92782115
0.636666667	0.921719485
0.64	0.915474234
0.643333333	0.909106947
0.646666667	0.902634039
0.65	0.896080358
0.653333333	0.889452666
0.656666667	0.882758519
0.66	0.876025592
0.663333333	0.869284365
0.666666667	0.862556365
0.67	0.855861696
0.673333333	0.8492094
0.676666667	0.842598933
0.68	0.836027318
0.683333333	0.829493624
0.686666667	0.822999436
0.69	0.816533359
0.693333333	0.810082289
0.696666667	0.803627202
0.7	0.79714736
0.703333333	0.790641713
0.706666667	0.78411834
0.71	0.777546729

0.713333333	0.77091506
0.716666667	0.764237251
0.72	0.757505536
0.723333333	0.750728907
0.726666667	0.743899705
0.73	0.737018684
0.733333333	0.730075332
0.736666667	0.723087019
0.74	0.716080301
0.743333333	0.709041788
0.746666667	0.70195427
0.75	0.694806644
0.753333333	0.687597597
0.756666667	0.680301115
0.76	0.672902146
0.763333333	0.66540815
0.766666667	0.657832213
0.77	0.650166935
0.773333333	0.642401288
0.776666667	0.634522518
0.78	0.626531873
0.783333333	0.61843221
0.786666667	0.610212336
0.79	0.601879176
0.793333333	0.593459063
0.796666667	0.584965003
0.8	0.576396183
0.803333333	0.567774739
0.806666667	0.559100604
0.81	0.550369013
0.813333333	0.541578134
0.816666667	0.532741405
0.82	0.52386083
0.823333333	0.514928512
0.826666667	0.505944894
0.83	0.496903246
0.833333333	0.48780361
0.836666667	0.47863993
0.84	0.469427887
0.843333333	0.460177979
0.846666667	0.450899429
0.85	0.441588434
0.853333333	0.43222878

0.856666667	0.422809397
0.86	0.413321068
0.863333333	0.403750194
0.866666667	0.394094538
0.87	0.384348339
0.873333333	0.374501174
0.876666667	0.36452771
0.88	0.35441243
0.883333333	0.344138759
0.886666667	0.333670037
0.89	0.322973629
0.893333333	0.312022601
0.896666667	0.300804374
0.9	0.289295358
0.903333333	0.277470171
0.906666667	0.265301539
0.91	0.252781927
0.913333333	0.239921017
0.916666667	0.226733414
0.92	0.213229736
0.923333333	0.19942832
0.926666667	0.185358608
0.93	0.171052414
0.933333333	0.156552101
0.936666667	0.141920191
0.94	0.127212596
0.943333333	0.112522635
0.946666667	0.097959773
0.95	0.083591524
0.953333333	0.069518611
0.956666667	0.055864278
0.96	0.042772631
0.963333333	0.030273971
0.966666667	0.018525303
0.97	0.007636302
0.973333333	-0.002310046
0.976666667	-0.011245128
0.98	-0.019098171
0.983333333	-0.025835367
0.986666667	-0.031434418
0.99	-0.035879139
0.993333333	-0.039187357
0.996666667	-0.041406539

1	-0.042605192
1.003333333	-0.042860257
1.006666667	-0.042269574
1.01	-0.040934119
1.013333333	-0.038970943
1.016666667	-0.036497307
1.02	-0.033627806
1.023333333	-0.030459257
1.026666667	-0.027108698
1.03	-0.023685886
1.033333333	-0.020247969
1.036666667	-0.016850628
1.04	-0.01355509
1.043333333	-0.010435793
1.046666667	-0.007432122
1.05	-0.00457931
1.053333333	-0.001882049
1.056666667	0.000663954
1.06	0.003084705
1.063333333	0.00534227
1.066666667	0.007471192
1.07	0.0095046
1.073333333	0.011461105
1.076666667	0.013357305
1.08	0.015217598
1.083333333	0.017059709
1.086666667	0.018889284
1.09	0.020715591
1.093333333	0.022534645
1.096666667	0.024344075
1.1	0.026144692
1.103333333	0.02793228
1.106666667	0.029698252
1.11	0.031438021
1.113333333	0.033139272
1.116666667	0.034790939
1.12	0.036387017
1.123333333	0.037922763
1.126666667	0.039391612
1.13	0.040791838
1.133333333	0.042117841
1.136666667	0.043363387
1.14	0.044523802

1.143333333	0.045594599
1.146666667	0.046573726
1.15	0.047459154
1.153333333	0.04824268
1.156666667	0.048920142
1.16	0.04948883
1.163333333	0.049939682
1.166666667	0.050271496
1.17	0.050482064
1.173333333	0.050566103
1.176666667	0.050522473
1.18	0.050360708
1.183333333	0.050089584
1.186666667	0.049713116
1.19	0.049232256
1.193333333	0.048649754
1.196666667	0.047971872
1.2	0.04720985
1.203333333	0.04637343
1.206666667	0.045473404
1.21	0.044519649
1.213333333	0.043522578
1.216666667	0.042491972
1.22	0.041436794
1.223333333	0.040367124
1.226666667	0.039290953
1.23	0.038212576
1.233333333	0.037135543
1.236666667	0.036062207
1.24	0.034991604
1.243333333	0.033925199
1.246666667	0.03287017
1.25	0.031827526
1.253333333	0.030798813
1.256666667	0.029786842
1.26	0.028796652
1.263333333	0.027828968
1.266666667	0.02688022
1.27	0.025946552
1.273333333	0.025028707
1.276666667	0.024128499
1.28	0.023248424
1.283333333	0.022390695

1.286666667	0.02155741
1.29	0.020748499
1.293333333	0.019963463
1.296666667	0.019201961
1.3	0.01846499
1.303333333	0.017753147
1.306666667	0.01706553
1.31	0.016400586
1.313333333	0.015758336
1.316666667	0.015137747
1.32	0.014538478
1.323333333	0.013961746
1.326666667	0.013407592
1.33	0.012873569
1.333333333	0.012358457
1.336666667	0.011860452
1.34	0.011379626
1.343333333	0.01091348
1.346666667	0.010459952
1.35	0.010016965
1.353333333	0.009583729
1.356666667	0.009160304
1.36	0.008745237
1.363333333	0.008337343
1.366666667	0.007935343
1.37	0.007538659
1.373333333	0.007147276
1.376666667	0.006762582
1.38	0.006385842
1.383333333	0.006018295
1.386666667	0.005660724
1.39	0.005313553
1.393333333	0.004977589
1.396666667	0.0046526
1.4	0.004338219
1.403333333	0.00403448
1.406666667	0.003740818
1.41	0.003456934
1.413333333	0.003182642
1.416666667	0.002917263
1.42	0.002660211
1.423333333	0.002410069
1.426666667	0.002165822

1.43	0.001926729
1.433333333	0.001692644
1.436666667	0.001463365
1.44	0.001238676
1.443333333	0.001018741
1.446666667	0.000803876
1.45	0.000594498
1.453333333	0.000390745
1.456666667	0.000192635
1.46	0
1.463333333	0
1.466666667	0
1.47	0
1.473333333	0
1.476666667	0
1.48	0
1.483333333	0
1.486666667	0
1.49	0
1.493333333	0
1.496666667	0
1.5	0
1.503333333	0
1.506666667	0
1.51	0
1.513333333	0
1.516666667	0
1.52	0
1.523333333	0
1.526666667	0
1.53	0
1.533333333	0
1.536666667	0
1.54	0
1.543333333	0
1.546666667	0
1.55	0
1.553333333	0
1.556666667	0
1.56	0
1.563333333	0
1.566666667	0
1.57	0

1.573333333	0
1.576666667	0
1.58	0
1.583333333	0
1.586666667	0
1.59	0
1.593333333	0
1.596666667	0
1.6	0
1.603333333	0
1.606666667	0
1.61	0
1.613333333	0
1.616666667	0
1.62	0
1.623333333	0
1.626666667	0
1.63	0
1.633333333	0
1.636666667	0
1.64	0
1.643333333	0
1.646666667	0
1.65	0
1.653333333	0
1.656666667	0
1.66	0
1.663333333	0
1.666666667	0
1.67	0
1.673333333	0
1.676666667	0
1.68	0
1.683333333	0
1.686666667	0
1.69	0
1.693333333	0
1.696666667	0
1.7	0
1.703333333	0
1.706666667	0
1.71	0
1.713333333	0

1.716666667	0
1.72	0
1.723333333	0
1.726666667	0
1.73	0
1.733333333	0
1.736666667	0
1.74	0
1.743333333	0
1.746666667	0
1.75	0
1.753333333	0
1.756666667	0
1.76	0
1.763333333	0
1.766666667	0
1.77	0
1.773333333	0
1.776666667	0
1.78	0
1.783333333	0
1.786666667	0
1.79	0
1.793333333	0
1.796666667	0
1.8	0
1.803333333	0
1.806666667	0
1.81	0
1.813333333	0
1.816666667	0
1.82	0
1.823333333	0
1.826666667	0
1.83	0
1.833333333	0
1.836666667	0
1.84	0
1.843333333	0
1.846666667	0
1.85	0
1.853333333	0
1.856666667	0

1.86	0
1.863333333	0
1.866666667	0
1.87	0
1.873333333	0
1.876666667	0
1.88	0
1.883333333	0
1.886666667	0
1.89	0
1.893333333	0
1.896666667	0
1.9	0
1.903333333	0
1.906666667	0
1.91	0
1.913333333	0
1.916666667	0
1.92	0
1.923333333	0
1.926666667	0
1.93	0
1.933333333	0
1.936666667	0
1.94	0
1.943333333	0
1.946666667	0
1.95	0
1.953333333	0
1.956666667	0
1.96	0
1.963333333	0
1.966666667	0
1.97	0
1.973333333	0
1.976666667	0
1.98	0
1.983333333	0
1.986666667	0
1.99	0
1.993333333	0
1.996666667	0
2	0

2.003333333	0
2.006666667	0
2.01	0
2.013333333	0
2.016666667	0
2.02	0
2.023333333	0
2.026666667	0
2.03	0
2.033333333	0
2.036666667	0
2.04	0
2.043333333	0
2.046666667	0
2.05	0
2.053333333	0
2.056666667	0
2.06	0
2.063333333	0
2.066666667	0
2.07	0
2.073333333	0
2.076666667	0
2.08	0
2.083333333	0
2.086666667	0
2.09	0
2.093333333	0
2.096666667	0
2.1	0
2.103333333	0
2.106666667	0
2.11	0
2.113333333	0
2.116666667	0
2.12	0
2.123333333	0
2.126666667	0
2.13	0
2.133333333	0
2.136666667	0
2.14	0
2.143333333	0

2.146666667	0
2.15	0
2.153333333	0
2.156666667	0
2.16	0
2.163333333	0
2.166666667	0
2.17	0
2.173333333	0
2.176666667	0
2.18	0
2.183333333	0
2.186666667	0
2.19	0
2.193333333	0
2.196666667	0
2.2	0
2.203333333	0
2.206666667	0
2.21	0
2.213333333	0
2.216666667	0
2.22	0
2.223333333	0
2.226666667	0
2.23	0
2.233333333	0
2.236666667	0
2.24	0
2.243333333	0
2.246666667	0
2.25	0
2.253333333	0
2.256666667	0
2.26	0
2.263333333	0
2.266666667	0
2.27	0
2.273333333	0
2.276666667	0
2.28	0
2.283333333	0
2.286666667	0

2.29	0
2.293333333	0
2.296666667	0
2.3	0
2.303333333	0
2.306666667	0
2.31	0
2.313333333	0
2.316666667	0
2.32	0
2.323333333	0
2.326666667	0
2.33	0
2.333333333	0
2.336666667	0
2.34	0
2.343333333	0
2.346666667	0
2.35	0
2.353333333	0
2.356666667	0
2.36	0
2.363333333	0
2.366666667	0
2.37	0
2.373333333	0
2.376666667	0
2.38	0
2.383333333	0
2.386666667	0
2.39	0
2.393333333	0
2.396666667	0
2.4	0
2.403333333	0
2.406666667	0
2.41	0
2.413333333	0
2.416666667	0
2.42	0
2.423333333	0
2.426666667	0
2.43	0

2.433333333	0
2.436666667	0
2.44	0
2.443333333	0
2.446666667	0
2.45	0
2.453333333	0
2.456666667	0
2.46	0
2.463333333	0
2.466666667	0
2.47	0
2.473333333	0
2.476666667	0
2.48	0
2.483333333	0
2.486666667	0
2.49	0
2.493333333	0
2.496666667	0
2.5	0
2.503333333	0
2.506666667	0
2.51	0
2.513333333	0
2.516666667	0
2.52	0
2.523333333	0
2.526666667	0
2.53	0
2.533333333	0
2.536666667	0
2.54	0
2.543333333	0
2.546666667	0
2.55	0
2.553333333	0
2.556666667	0
2.56	0
2.563333333	0
2.566666667	0
2.57	0
2.573333333	0

2.576666667	0
